# Actinomyces in the Bloodstream: A Pathogen or Passenger?

**DOI:** 10.7759/cureus.88001

**Published:** 2025-07-15

**Authors:** Zaraq R Khan, Lama Hanbali, Imad Majeed, Shruti Wadhwa, Anika Mehta, Forest W Arnold

**Affiliations:** 1 Infectious Diseases, University of Louisville Hospital, Louisville, USA; 2 Internal Medicine, University of Louisville Hospital, Louisville, USA; 3 Internal Medicine, University of Louisville School of Medicine, Louisville, USA; 4 Health Management, University of Louisville, Louisville, USA; 5 Infection Prevention and Control, University of Louisville Hospital, Louisville, USA

**Keywords:** actinomyces infection, antibiotic stewardship, bacteremia, gram-positive rods, normal flora

## Abstract

Background: *Actinomyces*, a genus of branching, gram-positive rods, is part of the normal flora in the oral cavity, genitourinary tract, and gastrointestinal tract. Despite their frequent isolation, advancements in molecular identification through matrix-assisted laser desorption/ionization time-of-flight mass spectrometry (MALDI-TOF MS) and 16S rRNA sequencing have complicated clinical interpretations, especially regarding their relevance when detected in blood cultures. Limited studies exist on *Actinomyces* bacteremia, leaving its clinical significance and treatment necessity largely uncertain. This study aimed to assess the clinical relevance of *Actinomyces* bacteremia and its impact on treatment decisions, with the goal of potentially minimizing unnecessary antibiotic use.

Methods: This cross-sectional, retrospective study was conducted using data on patients admitted to the University of Louisville Healthcare network between June 2022 and May 2023. Positive blood cultures for any species of *Actinomyces* were included, identifying 18 cases for analysis. Patient data was collected through chart reviews focusing on clinical presentations, treatment initiation, imaging, and follow-up outcomes for at least one month post-bacteremia. Data was kept confidential and securely stored, following ethical protocols.

Results: The mean age of patients was 58.5 years (SD = 21.26), with 66.67% male patients. Treatment was administered to only 11.11% (n=2) of patients, highlighting a predominant trend of non-treatment (n=16, 88.89%). Follow-up data for 17 cases indicated a mean duration of 6.58 months (SD = 6.15), ranging from 1 to 26 months. Mortality was observed in 33.33% of cases, although some deaths were attributed to unrelated causes.

Conclusion: *Actinomyces* bacteremia may often be clinically insignificant, as the majority of patients in this study did not receive treatment and showed varied outcomes. These findings suggest the need for a cautious approach in initiating prolonged antibiotic therapy for *Actinomyces* bacteremia.

## Introduction

*Actinomyces* is a genus of branching, gram-positive rods that constitute the normal flora of the oral cavity along with genitourinary (GU) and gastrointestinal (GI) tracts [1]. In the oral cavity, *Actinomyces* are known to initiate plaque development [2]. With the recent development of matrix-assisted laser desorption/ionization time-of-flight mass spectrometry (MALDI-TOF MS) and 16S rRNA sequencing, some studies have been able to identify at least 92 different species of *Actinomyces* [3]. Although recent advancements in molecular technology have helped in identifying various species, they have posed significant challenges to the healthcare community in ascertaining the clinical significance of the isolation of these bacteria from various clinical specimens, including blood cultures [4].

Cervicofacial, abdominopelvic, and pulmonary infections are the most prevalent presentations. Hematogenous spread is exceptionally rare and has been associated with *Actinomyces meyeri*, *Actinomyces israelii*, and *Actinomyces odontolyticus*. This infection typically affects individuals with poor oral hygiene, excessive alcohol consumption, immunosuppression, and underlying pulmonary diseases. The disease predominantly occurs between the second and sixth decades of life, with the peak incidence in the fourth and fifth decades [5]. The risk factors that predispose patients to develop bacteremia caused by *Actinomyces* are not fully elucidated; however, chronic sinusitis was identified as a risk factor in two of the cases of bacteremia. Diabetes mellitus is another prevalent comorbidity, particularly in adults who develop bacteremia [6,7]. *Actinomyces* species are genetically related to *Mycobacterium* and *Nocardia* species. Consequently, active infection, although uncommon, can mimic tuberculosis, nocardiosis, or malignancy, presenting a diagnostic challenge to physicians [6,8,9]. Males are disproportionately affected compared to females, exhibiting a ratio of 3:1, particularly among patients with low socioeconomic status [8]. Actinomycosis is usually treated for 6 to 12 months, depending on its extent [6].

Bacteremia and subsequent endocarditis due to *Actinomyces* species are rare, and since 1939, only 34 cases of *Actinomyces*-induced endocarditis have been reported [10,11]. With our thorough literature search, we were able to identify only four distinct observational studies that explore the clinical relevance of *Actinomyces* bacteremia [12-15]. The following were the study objectives: (1) to define the proportion of patients with *Actinomyces* bacteremia who were treated, and (2) to define the mortality of patients at follow-up. Our study aims to determine the clinical relevance of *Actinomyces* bacteremia and whether it consequently needs treatment, which, if not required, would potentially help to prevent the use of excessive antibiotics.

## Materials and methods

Study design and settings

This was a cross-sectional retrospective study. The patient population consisted of patients who were admitted or seen in the emergency room at the University of Louisville Healthcare network (UofL Health). All laboratory testing was performed at the University of Louisville Hospital, which served as the core laboratory for UofL Health. No outpatient clinic patients were included in our study.

Sample, sampling method, and size

A total of 18 patients were included in our study. A non-randomized consecutive sampling was done. Because of the rarity of *Actinomyces *bacteremia, all patients with a positive blood culture for any species of *Actinomyces *were included in our study.

Data collection

A search was performed using our microbiology database for positive cases starting from June 2022 to May 2023. A total of 18 patients were identified. As it was a retrospective study, a chart review of the patients was used for the assessment of relevant clinical signs, symptoms, imaging, decisions regarding treatment initiation, and follow-up. Patients were followed up for a minimum of one month with the help of chart review post-bacteremia for resolution of symptoms, recurrence of infection, and mortality.

Patient identifiable information, such as names and dates of birth, was collected as part of the study. This information was kept confidential and safe in a password-protected laptop, which could only be accessed by the primary investigator and collaborators. This data will be discarded after one year of collection.

Ethical consideration

Patient personal information was not shared or published in any way during our study. The University of Louisville Institutional Review Board (IRB) approved the study. All methods performed in this study were in accordance with the relevant guidelines and regulations of UofL IRB. Furthermore, the requirement for ethical approval for this study was waived by the UofL ethics committee. The need for informed consent was also waived by the UofL IRB ethics committee.

## Results

A total of 18 cases were analyzed across various variables. The mean age of participants was 58.5 years, with a standard deviation of approximately 21.26 years, indicating a range of 24 to 94 years. Gender distribution revealed 12 (66.67%) male patients and 6 (33.33%) female patients. In terms of treatment, 16 individuals (88.89%) did not receive treatment, whereas 2 individuals (11.11%) did receive treatment, indicating that most did not undergo treatment. Among the two patients who were treated, one had a polymicrobial peripheral intravenous line infection while the other had a dental abscess. *Actinomyces oris* was the most common isolated species, accounting for 11 out of 18 (61.1%), followed by *Actinomyces odontolyticus* (22.2%), while 3 never got speciated. The follow-up duration analysis for 17 observations indicated a mean follow-up of approximately 6.58 months, with a standard deviation of 6.15 months, and follow-up durations ranged from 1 to 26 months. Follow-up for one patient was not found in the chart review. Regarding mortality outcomes, 12 individuals (66.67%) survived, while 6 individuals (33.33%) died (Table 1).

**Table 1:** Patient data regarding positive blood cultures, species of actinomyces isolated from the blood cultures, and status of treatment along with related mortality and outcomes

Age (years)	Gender	No. of positive blood cultures	Species	Treated	Mortality	Follow-up (months)
65	M	1	Not specified	No	No	26
67	M	1	A. oris	No	No	12
87	M	1	A. oris	Yes	No	4
94	F	1	A. oris	No	Yes	4
68	M	1	A. oris	No	No	1
24	M	1	A. oris	No	No	9
37	M	1	A. odontolyticus	Yes	No	5
24	M	1	A. oris	No	Yes	N/A
38	M	1	A. odontolyticus	No	No	2
62	F	1	A. odontolyticus	No	Yes	1
58	M	1	A. oris	No	No	7
64	M	1	Not specified	No	No	4
36	M	1	A. oris	No	Yes	11
62	F	1	A. oris	No	No	10
74	M	1	A. oris	No	Yes	2
93	F	1	A. oris	No	No	5
52	F	1	Not specified	No	Yes	1
48	F	1	A. odontolyticus	No	No	8

## Discussion

The rarity of *Actinomyces* bacteremia raises questions about the clinical relevance of its presence in blood cultures. *Actinomyces* is typically part of the normal flora of the oral cavity, and GI and GU tracts [12]. *Actinomyces oris* was the most predominant isolated species in our cohort, accounting for 11 out of 18 cases; however, previous studies identified *Actinomyces odontolyticus* as their most isolated species [12,14]. Although our cohort showed a gender predominance, with 12 out of 18 patients affected being male, the literature varies on this. There does not seem to be a clear preference of* Actinomyces *to affect males over females, as one study in our literature review showed a male patient predominance [3,12], while the other showed a female patient predominance [14]. Typically, *Actinomyces* bacteremia does not require treatment, but when it does, the treatment is prolonged, anywhere between 6 and 12 months [6]. In our study, only two (11.11%) patients received treatment for their *Actinomyces *bacteremia, and follow-up of both patients showed clearance of the infection. In the six individuals who experienced mortality, all deaths were attributed to non-infectious causes. This suggests that *Actinomyces* in blood cultures may often be incidental findings rather than an indication of true infection [14]. This aligns with our hypothesis that *Actinomyces *bacteremia should not require treatment in many cases, in contrast with other pathogenic bacteria found in blood cultures. This finding is also consistent with previous reports on the presence of *Actinomyces* species in blood cultures. Jeffery-Smith et al. (2016) found that only 10 of 60 *Actinomyces* spp. in blood cultures required treatment, and most of them had recognized clinical risk factors [12]. In another study, Datta et al. (2017) treated 6 out of the 21 patients with *Actinomyces* bacteremia with prolonged antibiotics [14]. Similarly, these studies, along with our results, support the hypothesis that *Actinomyces* spp. in the blood may be a more incidental than pathogenic finding.

The causes of mortality in our cohort were not necessarily infectious in origin. One patient developed small bowel obstruction at the four-month follow-up that eventually led to acute respiratory failure. He was also deemed to have septic shock; the cause was unknown, and he required vasopressor support. The family eventually chose comfort care for him. The second patient died during the same hospitalization due to cardiac failure secondary to refractory atrial fibrillation. The third patient had nonalcoholic steatohepatitis (NASH) cirrhosis and end-stage renal failure, due to which she chose comfort care at the one-month follow-up, resulting in the death of the patient. The fourth patient died as per the chart check, but we could not find the exact reason for his death. He had a significant history of substance use disorder with psychotic symptoms, with multiple previous admissions for suicidal ideation; however, he was doing fine from an infection standpoint at the seven-month follow-up. The fifth patient died due to complications of a head injury secondary to a fall at the two-month follow-up. The sixth patient died during the same hospitalization due to sepsis, with blood cultures positive for methicillin-resistant *Staphylococcus aureus*.

The important clinical concern at this point is to seek out the causality of this transient bacteremia. There is a certain possibility that this may represent transient bacteremia brought on by a minor/routine procedure, such as a colonoscopy or dental manipulation, and that just happened to be captured at the time blood cultures were drawn [12]. Certain studies have also revealed that *Actinomyces *may be part of the skin flora, which may explain cultures being contaminated while the blood is drawn in case standard antiseptic protocols are not followed [16]. Advancements in clinical technologies, like MALDI-TOF MS and 16S rRNA sequencing, have improved the identification of *Actinomyces *species that previously were regarded as skin contaminants like *Corynebacterium* [17]. Ultimately, while these technologies enhance the sensitivity of bacterial identification, they increase challenges in discriminating incidental findings from true clinically relevant infections. This makes it difficult to manage the bacteremia because if a true bacteremia is left untreated, it can be fatal.

This study has several limitations that impact its generalizability. The small sample size and single-center design restrict the applicability of the findings to broader populations. As a retrospective analysis, the study was susceptible to missing or incomplete data, and lacks a control group for comparative analysis. In several instances, *Actinomyces* species were not identified, hindering the assessment of species-specific clinical relevance. The source of bacteremia was frequently unclear, and the possibility of blood culture contamination cannot be entirely ruled out. Furthermore, the follow-up durations were heterogeneous, and one patient had no follow-up data, which may influence the evaluation of long-term outcomes.

## Conclusions

This study aimed to highlight the need to re-evaluate this bacterium's clinical significance and management. In the study cohort, the majority of patients did not receive treatment for their *Actinomyces *bacteremia, and although outcomes varied, mortality was largely attributable to non-infectious causes. This strongly indicates that the presence of *Actinomyces* in blood cultures may simply represent an incidental finding rather than an indication of true infection requiring prolonged antibiotic treatment. We recommend a more judicial approach to treating *Actinomyces* bacteremia, which can ultimately reduce unnecessary antibiotic use and its associated risks.
